# Computational fluid dynamics for vascular assessment in hepatobiliopancreatic surgery: a pilot study and future perspectives

**DOI:** 10.1007/s00464-025-11536-4

**Published:** 2025-04-01

**Authors:** Carolina González-Abós, Roberto Molina, Sofía Almirante, Mariano Vázquez, Fabio Ausania

**Affiliations:** 1 HBP and Liver Transplant Surgery Department, Hospital Clínic de Barcelona, Barcelona, Spain; 2 ELEM Biotech, Barcelona, Spain; 3 Gene Therapy and Cancer, Instituto de Investigaciones Biomédicas August Pi I Sunyer (IDIBAPS), Barcelona, Spain; 4 Universitat de Barcelona, Barcelona, Spain

**Keywords:** Computational fluid dynamics, Blood flow prediction, Pancreatic surgery, Preoperative planning

## Abstract

**Introduction:**

In major hepatobiliopancreatic surgery, an accurate preoperative planning is essential. Postoperative impaired blood supply due to arterial disease or variants can cause postoperative complications. Computational fluid dynamics has previously been successful in revealing distinct features of haemodynamic disturbances. The purpose of our study is to describe the feasibility of a computational fluid dynamics model to predict hepatic artery flow and its variations following gastroduodenal (GDA) or common hepatic (CHA) artery ligation.

**Material and methods:**

This is a pilot study including 20 patients undergoing robotic pancreaticoduodenectomy at a single centre. Preoperative images and intraoperative vascular flows were used to the computational model. Three scenarios of the hepatic artery were analysed: (1) without any clamps, (2) clamped GDA and (3) clamped CHA. Patients 1 to 15 were used to develop the model, and patients 15 to 20 were used for model validation. Finally, the model was tested in 3 abnormal cases: celiac trunk stenosis (2) and replaced right hepatic artery (1).

**Results:**

The selected methodology proved to be reproducible, with the CFD model demonstrating 100% accuracy in predicting blood flow redistribution after gastroduodenal artery (GDA) clamping and 80% accuracy following common hepatic artery (CHA) clamping. The model accurately simulated reversed GDA flow in cases of celiac trunk stenosis and displayed independent flow distribution in patients with anatomical variations, even without prior specific model training.

**Conclusion:**

The developed computational model accurately predicts flow variations in the proper hepatic artery in case of gastroduodenal artery and common hepatic artery clamping. Further studies are needed to validate this methodology.

**Supplementary Information:**

The online version contains supplementary material available at 10.1007/s00464-025-11536-4.

Major hepatobiliary and pancreatic (HPB) surgeries are mostly complex procedures associated with high morbidity. An accurate preoperative planning is essential for allowing a safe surgery in these patients. Knowledge of arterial anatomy and its variations is a crucial prerequisite to perform safe HPB surgery [1], and only approximately 55% of patients show a normal anatomy according to Michels classification [2]. Blood supply to abdominal organs mainly originates from the celiac trunk (CT) and superior mesenteric artery (SMA) [3]. The use of last-generation multidetector computer tomography (MDCT) allows high-quality assessment of vascular anatomy and its variations [4]. However, arterial diseases such as stenosis and/or arteriosclerosis can lead to altered blood supply. Pancreatic surgery requires the interruption of some of the arteries arising from CT and SMA [5]. Pancreaticoduodenectomy (PD) implies gastroduodenal (GDA) and inferior pancreaticoduodenal (IPDA) arterial ligation, and these arteries represent the most important communication between CT and SMA arterial systems: an arterial insufficiency of one system can be counterbalanced by hypertrophy of the other one: for this reason, many significant CT stenoses can be undiagnosed before surgery. In fact, up to 10% of patients undergoing PD show a CT stenosis, and in these patients GDA ligation can lead to increased major complications, including biliary and liver ischaemia [6]. Preoperative MDCT can detect a significant CT stenosis with high sensitivity [7]; however, the haemodynamic impact of the stenosis is difficult to predict. Doppler ultrasound can contribute to a better assessment, allowing the measurement of CT and common hepatic artery (CHA) blood flow, although its feasibility is limited by patient characteristics such as obesity, anatomical variability and the difficulty of assessing smaller calibre arteries such as the GDA [7]. 4D flow magnetic resonance allows comprehensive in vivo measurement of three-dimensional blood flow dynamics in large vessels, but its accuracy in small and tortuous vessels, such as the CHA, is reduced due to blood turbulence [8]. None of the previous technologies provide any information on the effect of GDA ligation. Besides that, in some exceptional cases, CT resection is needed for treatment of a pancreatic body neoplasm that affects CT, in these cases prediction of future blood flow from the GDA to the liver is needed [9]. Arteriography with hepatic artery occlusion is the only test that can be helpful to predict the haemodynamic effect of GDA ligation or CHA ligation. However, this is an invasive test with a non-negligible risk of complications [10]. Other technologies, such as dynamic contrast-enhanced CT or PET/CT, have been used to assess organic perfusion in the brain, but have not been studied in the abdomen due to its complex anatomy [11].

Computational fluid dynamics (CFD) has previously been successful in revealing distinct features of haemodynamic disturbances in cardiovascular diseases such as aortic aneurysms and dissections [12–14]. CFD has also been used for studying blood flow distribution in portal vein and hepatic artery [15, 16]. CFD enables the study of various conditions that may not be present in the current images by modifying the geometry of the model. This flexibility allows for the simulation and analysis of hypothetical scenarios and the impact of different pathological changes on blood flow dynamics [17]. The purpose of our study was to describe the feasibility of a computational fluid dynamics model that can preoperatively predict the arterial flow of proper hepatic artery (PHA) and the flow variations following GDA or CHA ligation in patients undergoing pancreatic surgery.

## Material and methods

### Patients

We conducted a prospective analysis of 20 patients undergoing pancreaticoduodenectomy from January 2023 to January 2024, at a single institution. The inclusion criteria were as follows: (a) any indication for pancreaticoduodenectomy, (b) Michels Type I arterial hepatic anatomy, (c) absence of celiac trunk and superior mesenteric artery stenosis at preoperative CT scan and (d) informed consent. Exclusion criteria include: (a) poor quality images, (b) arterial abnormalities, such as infiltration of the arteries included in the segmentation, (c) intraoperative inability to measure blood flow. Surgical procedure was performed as previously defined by our centre and the surgical approach was recorded for each patient [18], including open and robotic approaches. The common hepatic artery (CHA) and proper hepatic artery (PHA) at the bifurcation of the gastroduodenal artery (GDA) were selectively studied to create this computational model.

Additionally, the developed model was tested in three new patients: two with radiologically significant celiac trunk stenosis and one patient with a replaced right hepatic artery (RHA) from superior mesenteric artery (SMA).

### Flow parameter acquisition

**Fig. 1:**
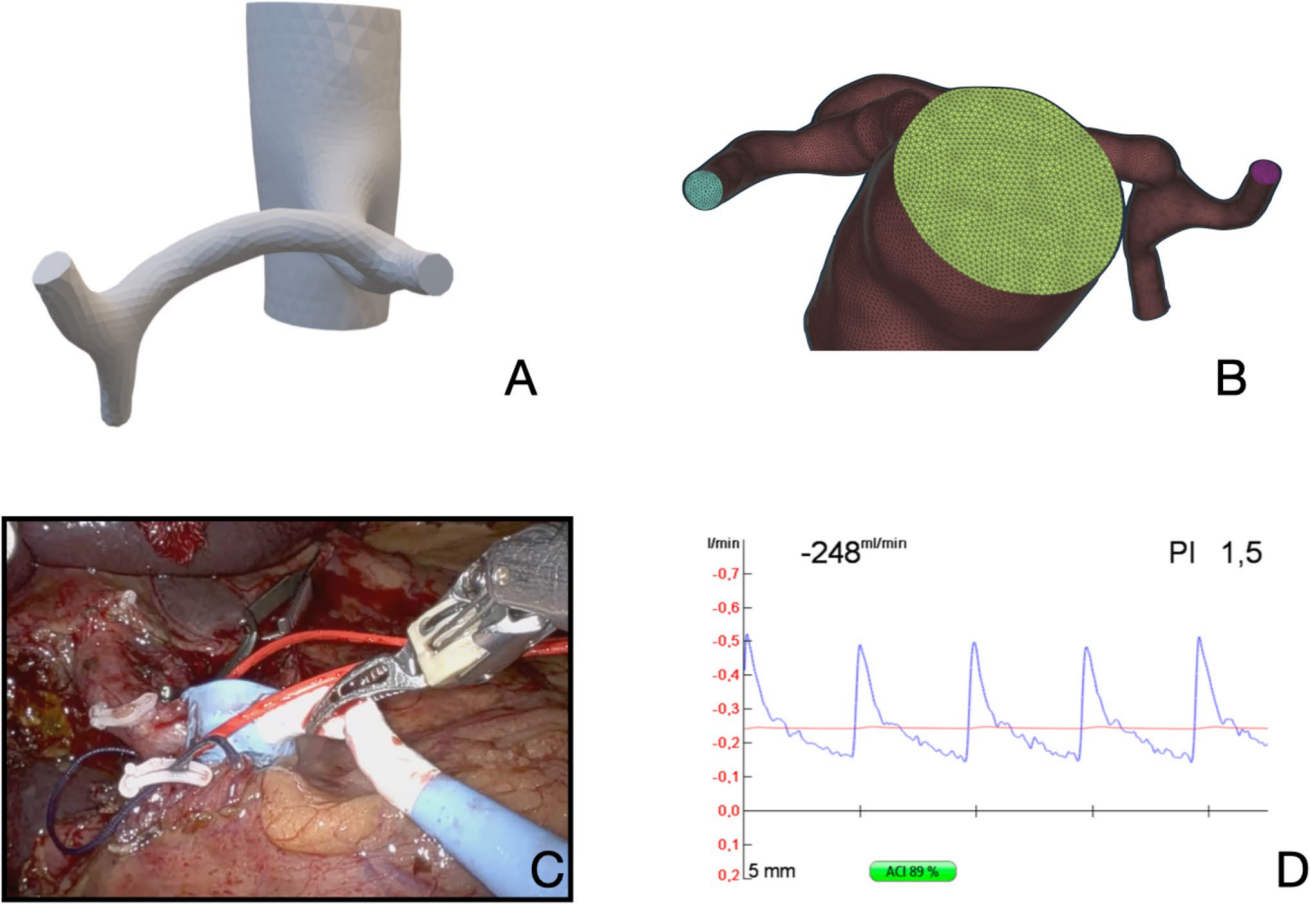
Shows image segmentation, meshing of the segmented anatomy and intraoperative blood flow measurement. These are necessary steps to develop the defined computational model. **A** Segmentation of the CT, CHA, PHA and GDA starting from the aorta; **B** Section of the mesh displaying distinct colours representing different labels, accompanied by visible perpendicular cuts at the exits. Celiac trunk in yellow, splenic artery in blue and proper hepatic artery in magenta; **C** Intraoperative blood flow measurement of hepatic common artery with MiraQ ™ Vascular and a 5 mm probe during a robotic pancreaticoduodenectomy; **D** Blood flow waveforms obtained from intraoperative measurement with MiraQ™ Vascular. An ACI > 50% indicates good performance of the flow measurement

Intraoperative real-time blood flow measurement was obtained during PD with MiraQ ™ Vascular device and Medistim Vascular TTFM Probes (MEDISTIM). Probe size was selected according to intraoperative size of CHA and GDA, generally 5 to 8 mm probes were used. All values were obtained in presence of haemodynamic stability and absence of vasoactive drugs and, in order to avoid physiological variability, blood flow was measured for 20 s at a time and the annotated value was the mean blood flow during these 20 s, including both respiratory phases. After dissecting hepatic hilum, transit-time ultrasound flowmeter was used (Fig. 1C), and flow curves were recorded, seen Fig. 1D. First, PHA, CHA and GDA flows were measured in baseline conditions. Subsequently, GDA was temporally clamped and PHA and CHA flows were measured (scenario 1). Finally, CHA was temporally clamped and PHA and GDA flows were measured (scenario 2). Subsequently, GDA was divided, and the surgical procedure was completed. Blood flow measurement variability could show an error margin between 10 and 15% according to the intraoperative conditions and probe position against the vessel, as defined by MEDISTIM.

## Methods overview for computational fluid dynamics (CFD) analysis

### 3D model generation

Patients’ arterial phase images from MDCT in DICOM format were used to create 3D models. Segmentation of the arteries (CHA, CT, GDA and PHA) was done using 3D-Slicer, an open-source platform for medical image analysis [19]. Semi-automatic segmentation was applied to extract the targeted arteries’ geometries (Fig. 1A).

### Meshing process

After segmentation, the arterial geometries were reconstructed, and a mesh was generated using ANSA, a computer-aided engineering (CAE) software [20]. Maximum mesh length was set at 0.3 mm for small arteries and 0.7 mm for the aorta to ensure high-quality meshes (Fig. 1B).

### Boundary conditions and CFD model

Three meshes were created per case. Haemodynamic modelling was based on the two-parameter Windkessel (WK) model, known for simulating realistic aortic pressures and flows [21]. WK parameters were tuned using patient-specific arterial flow measurements. GDA clamping was simulated by setting zero flow at its outlet. Simulations were performed using Ayla, a high-performance computational platform [22]. For simplicity, constant flows were used instead of pulsatile flows, reducing computation time and model complexity. Arterial pressure was not measured due to system constraints.

### CFD model validation

Patient-specific variables were used to infer initial arterial flow without direct measurements. The predicted flows were validated through simulations in five new patients. GDA and CHA clamping scenarios were simulated, comparing expected and actual PHA flows. Predictions were considered accurate when the real PHA flow fell within the predicted flow range.

### Pancreaticoduodenal arcade simplification

To reduce computational complexity, the pancreaticoduodenal arcade was simplified by connecting the distal GDA and inferior pancreaticoduodenal artery. Resistances were adjusted accordingly.

### Fluid mechanics analysis

Simulations were conducted for open, GDA-clamped and CHA-clamped scenarios. The first 15 patients were used to calibrate the computational model, while patients 16 to 20 were used for validation. WK parameters were adjusted iteratively based on blood flow measurements. Aortic pressure was fixed at 100 mmHg, although adjusting it proportionally would not change flow distribution due to resistance adjustments. Simplified representations of pancreaticoduodenal arcades further reduced computational costs.

The model was also tested in two patients with celiac trunk stenosis and one patient with a replaced right hepatic artery (Michel’s Type 8), confirming its applicability to varied anatomical conditions.

## Results

### Patient’s characteristics

Finally, 23 patients were included in the study. One patient was excluded from the analysis due to poor quality of CT images that did not allow an accurate segmentation. Two additional patients were excluded due to extremely low GDA flow and GDA tumoral infiltration, arterial infiltration was detected when reviewing the CT images postoperatively. Table S1 shows intraoperative collected patient data of this patients 1 to 15. Intraoperative flow measurements were in line with the physical expected behaviour of blood flow following the Principle of continuity. Initial CHA flow decreases when GDA is clamped (scenario 1), whereas PHA flow increases (Fig.S1). Additionally, the model was tested in two patients with radiologically relevant celiac trunk stenosis and one patient with a replaced right hepatic artery (RHA) from superior mesenteric artery (SMA).

### Fluid mechanics simulations

The selected Windkessel (WK) parameters produced reliable and reproducible results. Simulated blood flow closely matched intraoperative measurements, demonstrating the model’s accuracy. Simulated arterial wave pulses were comparable to real arterial waveforms recorded during surgery, including both systolic and diastolic peaks. Additionally, the simulations showed lower velocities near the arterial walls, consistent with physiological flow patterns observed in clinical settings.

### Model adjustment: open configuration

**Fig. 2:**
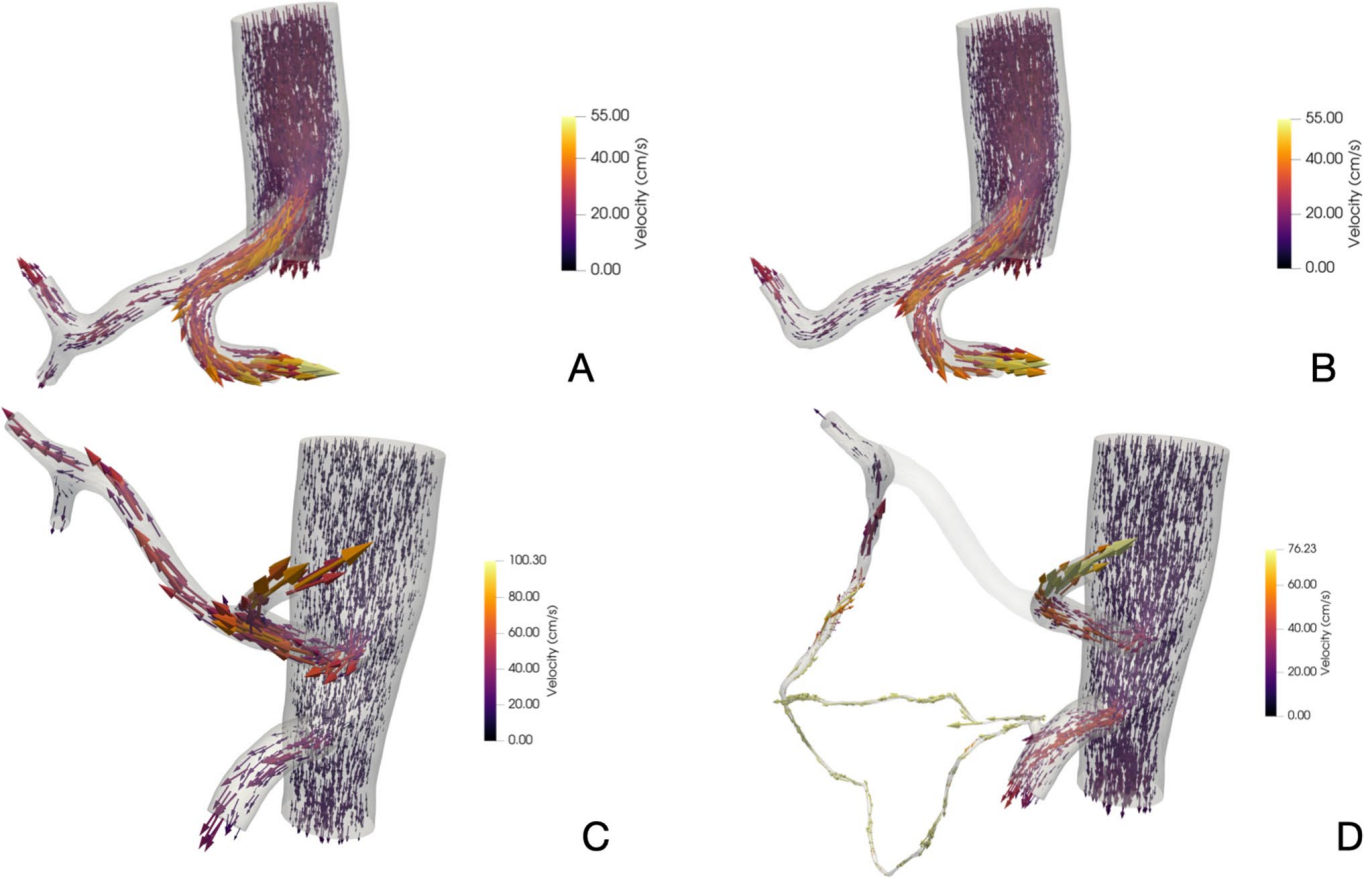
Computational fluid dynamics simulation of patient P18 in scenario 1 and patient P16 in scenario 2. **A** Simulation of blood flow distribution in initial configuration of patient P18; **B** Simulation of blood flow distribution after GDA clamping (scenario 1) of patient P18; **C** Simulation of blood flow distribution in initial configuration of patient P16; **D** Simulation of blood flow redistribution after CHA clamping (scenario 2) of patient P16

Patients 1 to 15 were used to adjust the model. In patients without CT stenosis the simulation showed that the fluid followed the expected path, pumping blood from CHA to GDA and its arcade; Fig. 2A shows the CFD model in the initial anatomical configuration. The model was considered to be well-calibrated when the difference between the flow measured during surgery and the simulated flow was less than 1%, which is negligible compared to the error margin of the probes used, typically between 10 and 15%.

### Simulation scenarios and anatomical variations

Simulation Scenario 1: Clamped Gastroduodenal Artery (GDA). The CFD model was calibrated using data from patients 1 to 15, while patients 16 to 20 were used for validation. Table 1 summarises the predicted PHA flow increase after GDA clamping, showing a relative error of less than 10%. This margin of error is likely due to inherent variability in flow measurements. The model demonstrated 100% accuracy, as real PHA flows after GDA clamping consistently fell within the expected ranges provided by the simulations. In all cases, measured PHA flow exceeded the predicted minimum, ensuring that the liver’s inflow was not underestimated, thus reducing potential postoperative risks. Figure 2B illustrates the new arterial geometry after GDA clamping, with velocity patterns aligning closely with realistic clinical flow behaviour.

**Table 1:** Shows predicted PHA flow increase after GDA clamping in patients 16 to 20

ID	CHA (ml/min)	GDA (ml/min)	PHA0* (ml/min)	Real PHA1 (ml/min)	Predicted PHA1 (ml/min)	Error	Expected range (min PHA1)	Expected rang (max PHA1)	Rel. error of min
ID16	331	60	271	314	289,2	8%	247,1	331,3	21%
ID17	144	18	126	120	129,6	5%	112,5	146,7	6%
ID18	179	52	127	136	130,8	4%	105,1	156,6	22%
ID19	215	54	161	153	171,6	3%	142,3	201,2	7%
ID20	213	67	146	176	159,6	9%	128,25	190,95	27%

The error is calculated with the difference between predicted and real PHA1 over real PHA. Relative error over the minimum expected value = (realPHA1-min PHA1)/real PHA1. ID, identification number; CHA, measured initial common hepatic artery flow; GDA, measured initial gastroduodenal artery flow, PHA0*; inferred initial proper hepatic artery flow, PHA1; proper hepatic artery flow after GDA clamping

**Fig. 3:**
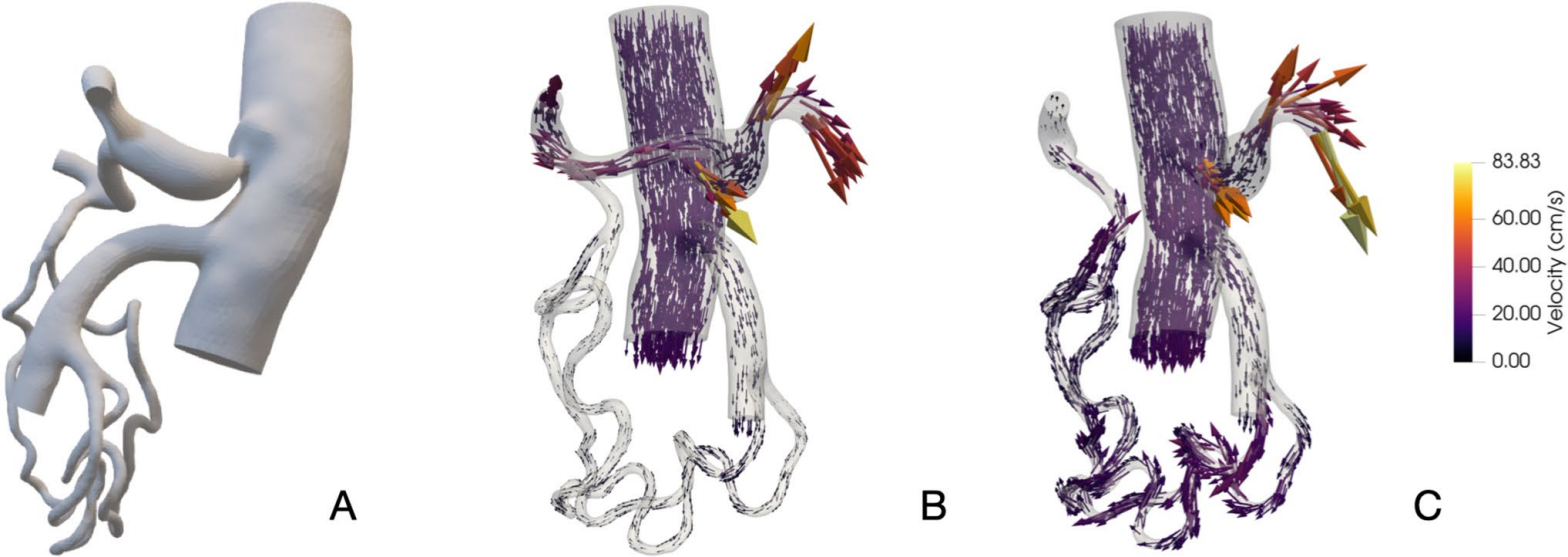
Computational fluid dynamics simulation in patient with a clinically relevant celiac trunk stenosis. **A** Model segmentation of patient 21, showing radiologically significant celiac trunk stenosis; **B** Initial configuration, CHA and GDA are open. Flow redistribution due to CT stenosis causes the flow of the gastroduodenal artery to be reversed and go towards the liver; **C** Scenario 2, CHA is clamped. GDA flow to the liver is higher than in patients without CT stenosis. Approximately 150 ml/s are expected

Simulation Scenario 2: Clamped Common Hepatic Artery (CHA). The anatomical complexity in the pancreatic head region and the non-hypertrophic pancreaticoduodenal arcade made segmentation challenging, increasing the computational cost. Despite these difficulties, the model produced accurate predictions. Patients 1 to 15 were used for parameter adjustments, while patients 16 to 20 were evaluated for validation. Table 2 shows that the model achieved 80% accuracy in predicting PHA flow after CHA clamping, with errors mainly attributed to segmentation challenges. Figure 3A, B depicts the new arterial geometry and flow pattern, while Fig. 3C illustrates blood flow redistribution after CHA clamping (Fig. 3).

**Table 2:** Shows predicted PHA flow increase after CHA clamping in patients 16 to 120, excluding patient 15

ID	CHA (ml/min)	PHA0* (ml/min)	GDA (ml/min)	Real PHA2 (ml/min)	PHA2 pred (ml/min)	Predicted expected range (ml/min	Error
ID16	331	271	60	91	103,2	89,72–118,7	13,1%
ID17	144	126	18	43	47,5	42,9–51,06	10,4%
ID18	179	127	52	203	74,5	68,9–80,8	63,3%
ID19	215	161	54	98	102,3	94,3–109,9	4,1%
ID20	213	146	67	99	106,2	98,2–114,1	8,1%

The error is calculated with the difference between predicted and real PHA2 over real PHA. ID, identification number; CHA, measured initial common hepatic artery flow; GDA, measured initial gastroduodenal artery flow, PHA0*; inferred initial proper hepatic artery flow, PHA2; proper hepatic artery flow after CHA clamping

**Fig. 4:**
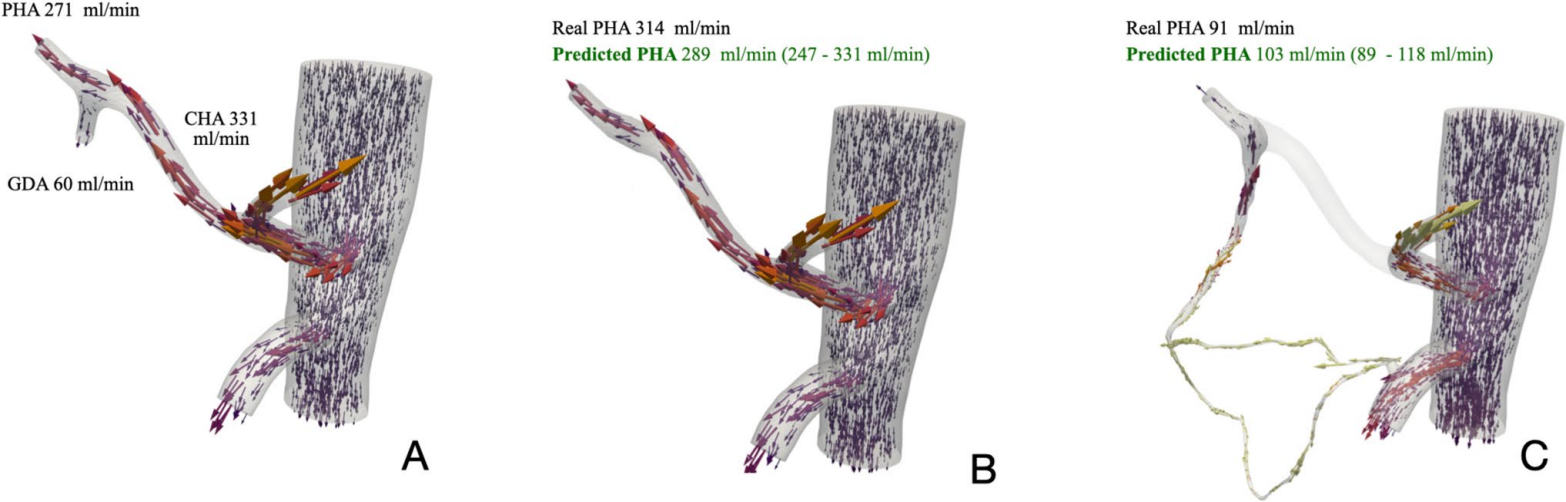
Comparison of real and predicted PHA blood flow for patient 16. **A** Simulation of blood flow distribution in the initial configuration of patient P16 with quantitative flows from intraoperative measurement; **B** Comparison between intraoperative measured PHA blood flow (314 ml/min) and predicted PHA blood flow (247–331 ml/min) after GDA clamping (scenario 1); **C** Comparison between intraoperative measured PHA blood flow (91 ml/min) and predicted PHA blood flow (89–118 ml/min) after CHA clamping (scenario 2)

Figure 4 shows the comparison of real PHA blood flow and predicted PHA blood flow in both scenarios.

### Anatomical variations

The CFD model successfully simulated patients with anatomical variations. Patient 23, with a replaced right hepatic artery (RHA) originating from the superior mesenteric artery (SMA), demonstrated accurate flow redistribution predictions. After GDA clamping, the liver received blood primarily from the celiac trunk, with minimal redistribution in the accessory hepatic ducts (AHD). The predicted RHA blood flow was 59 ml/min, closely matching the intraoperative measurement of 67 ml/min (range: 56.95–77.05 ml/min), considering a 15% variability in the measurement device. Figure 4 highlights these flow patterns.

### Celiac trunk stenosis

Two patients with celiac trunk stenosis were also modelled using geometries extracted directly from MDCT scans. The resulting CFD models revealed distinct blood flow distributions compared to other patients, with the GDA becoming the primary source of liver perfusion rather than the CHA. Figure 3 illustrates blood flow redistribution in a patient with clinically significant celiac trunk stenosis, highlighting the unique haemodynamic adjustments caused by this condition.

## Discussion

In this study we developed a computational model of the common hepatic artery at the bifurcation with the gastroduodenal artery based on CT scan data from 20 patients undergoing pancreaticoduodenectomy and intraoperative blood flows. This is the first study to demonstrate the feasibility of the developed methodology to assess hepatic arterial flow of the hepatic artery in patients without arterial variations.

Previous authors have shown that computational flow analysis can reveal distinct features of haemodynamic disturbances in the aorta [11]. In coronary assessment, computational flow analysis from MDCT showed the highest diagnostic performance for vessel-specific ischaemia in patients with coronary disease [23], presenting computational flow analysis as a good alternative in non-invasive blood flow assessment. CFD has also been used to analyse flow distribution in the branches of the hepatic artery and portal blood flow redistribution after major hepatectomy in a small cohorts of patients, but they did not simulate conditions other than those shown by the CT scan [24, 25].

Predicting future blood supply to abdominal organs is of paramount importance, especially in major HPB surgery, because hepatic artery insufficiency caused by GDA ligation is associated with clinically relevant pancreatic fistula, liver perfusion failure, gastric ischaemia, clinically relevant biliary leakage and the need for intensive care [6]. The presence of arterial disease in patients undergoing surgery is a current concern as more elderly patients are considered for aggressive surgery and the presence of metabolic syndrome is increasing in the general population [26]. Although the presence of arterial calcifications or stenosis at preoperative MDCT is common, their haemodynamic impact is unknown, and it is difficult to predict without an invasive test such as arteriography.

We have developed a methodology to predict hepatic arterial flow under normal conditions to provide a novel approach to this problem. To achieve this, the current study analyses the common hepatic artery (CHA) and the proper hepatic artery (PHA) located at the bifurcation of the gastroduodenal artery (GDA). The intraoperative data measured in the patients showed that the studied arterial system behaves as expected by the laws of fluid biophysics (Table S1). On one hand, the initial CHA flow decreases when GDA is clamped (scenario 1) because the total flow coming from the aorta is redistributed to the remaining open arterial branches of the entire abdominal aorta. Nevertheless, the flow in the PHA after GDA clamping increases compared to the initial PHA flow due to the flow redistribution. On the other hand, intraoperatively, the reversion and flow magnitude of the GDA after CHA clamping showed the relevance of analysing the communication between these two arterial systems when assessing supramesocolic arterial vascularisation. This compliance with physical laws allows us to trust the development of a model based on the same laws. The developed methodology has demonstrated reproducibility, and the current model has shown an acceptable accuracy in the different study scenarios. This pilot study allowed us to define resistances and WK parameters for haemodynamic modelling of the anatomical region under study. The developed CFD model can predict the flow distribution in the study area in initial anatomy, scenario 1 and scenario 2, showing the minimum expected arterial blood flow. Physical laws are not able to fully explain the increase in PHA blood flow after GDA clamping, as in all cases, the real increase in blood flow is higher than the predicted. In our opinion, the most likely explanation for the higher increase in blood flow in the PHA after GDA clamping can be related to the hepatic arterial buffer response (HABR). The HABR is a regulatory mechanism that allows to maintain a relatively constant value of hepatic blood flow, in case of a decrease of the hepatic portal blood flow, the total hepatic blood flow is, in part, restored by an increase in HA blood flow [27]. Accordingly, once the GDA is clamped, blood flow distributed through the pancreaticoduodenal arcade decreases, reducing the blood that is subsequently drained through the portal vein system. This decrease in hepatic portal vein blood flow can activate the HABR, increasing CHA blood flow. This increase is added to the expected blood flow redistribution explained by physical laws, as commented earlier. Based on this argument, by adjusting the parameters in this scenario, the model can be adapted to the expected biological behaviour, showing a reproducible blood flow behaviour in all cases. Despite that, being able to predict the minimum expected blood flow after GDA clamping shows the model as a safe technology for preoperative planning. According to our results, PHA flow after GDA section will be always higher than the minimum expected flow by the CFD model, so using this predictive model we can ensure an adequate liver arterial blood supply. The model was also tested when clamping CHA, showing good results. However, segmentation of the pancreaticoduodenal arcade was difficult and improvements in this simplification are needed to ensure data accuracy. After CHA clamping, the CFD model performed better in patients who showed some degree of pancreaticoduodenal arcade hypertrophy, probably due to the easiness of model building due to a better CT images interpretation. Probably, other patient variables and radiomics features should be taken in consideration to improve the accuracy of the prediction in this case scenario, reducing the impact of the anatomical simplification of the segmentation. Nevertheless, the use of CFD in CHA clamping would be of interest especially in cases of pathological infiltration of the CT on in case of CT stenosis, which are usually associated with hypertrophy of the pancreaticoduodenal arcade. The model has also been reported to perform well in pathological conditions and in case of anatomical variations. One patient with a replaced RHA from the SMA was studied and the blood flow redistribution was as expected. In the case of the replaced RHA, GDA clamping had lower effect in CHA flow, probably due to the continued flow of RHA coming from SMA. When analysing the patient with a clinically significant celiac trunk stenosis, the 3D reconstruction showed a higher GDA section compared to non-pathological CT scans. By combining the higher GDA area with the reduced CT area, the model autonomously simulated an initial GDA blood flow directed to the liver. This reversal of the blood flow as shown by the computational model confirms that CFD is able to interpret the CT stenosis as an anatomical variation that affects the redistribution of the blood flow. It was not necessary to modify the model in order to obtain these results. Therefore, it can be assumed that the developed model is capable of simulating different conditions than those used for its development.

The main limitation of this study is the small number of patients analysed. However, this is a feasibility study and based on previous computational tests, an accurate similarity to real life conditions was obtained with this sample size, providing us with a first computational model with high accuracy.

The primary objective of this study was to validate the methodology used to develop the computational fluid dynamics (CFD) model and ensure its reproducibility. The model demonstrated high accuracy, though further analysis with a larger patient cohort is needed to assess its external validity. Additionally, future studies should focus on refining the initial flow inference of the common hepatic artery (CHA) by identifying key patient characteristics that could enhance the estimation of initial flows without requiring intraoperative measurements.

The model’s performance after CHA clamping was less accurate in cases involving complex segmentation of the pancreaticoduodenal arcade. Simplifying the arcade in the model may impact reliability; however, complete segmentation is often unfeasible due to anatomical complexities, particularly when arteries from the celiac trunk (CT) and superior mesenteric artery (SMA) converge in the pancreatic head. Moreover, the computational cost of full segmentation would be prohibitively high. In such cases, the simplified model remains a practical approach.

The assumption of standard blood pressure and continuous flow does not seem to compromise the model’s validity. Since blood pressure remains consistent between the model’s inlet and outlets, its effect on blood velocity and flow redistribution should be negligible. While incorporating pulsatile blood flow could help detect arterial abnormalities or altered liver parenchyma resistance, doing so would require dynamic CT scans, leading to computational costs that are currently impractical. Furthermore, in non-cirrhotic patients, pulsatile flow would likely provide limited additional information.

Although the model has been tested in three patients with abnormal conditions, further validation in a broader cohort of patients with hepatic artery variants and celiac trunk stenosis is necessary. Despite these limitations, initial results are encouraging and suggest the model’s potential for broader clinical applications.

The next phase of this project will involve expanding the simulated area to fully encompass the celiac trunk (CT) and superior mesenteric artery (SMA). This will allow for a more detailed analysis of the bidirectional interactions between these two arterial systems. Incorporating additional vessels such as the SMA and collateral branches will enable the model to better capture the complexity of blood flow distribution and flow variations, particularly in the proper hepatic artery (PHA) and flow reversions in the gastroduodenal artery (GDA). This expanded vascular representation could significantly enhance the understanding of flow dynamics in these interconnected systems.

Further improvements will involve integrating patient-specific variables and radiomics features to refine the model’s accuracy, especially in cases where MDCT image segmentation is challenging. Once the CFD model methodology is fully validated, efforts will focus on adapting the model for cirrhotic patients by including liver resistance and portal blood flow dynamics.

In conclusion, the developed computational model effectively predicts blood flow variations in the common hepatic artery (CHA) and PHA at the GDA bifurcation, a critical anatomical landmark in major liver and pancreatic surgeries. Future studies are essential to validate the methodology further. Expanding the study area will facilitate the development of a comprehensive model capable of preoperatively assessing arterial abnormalities and flow redistribution in the vascular arcades connecting the CT and SMA.

## Supplementary Information

Below is the link to the electronic supplementary material.

Supplementary file1 (DOCX 20 KB)

Supplementary file2 (PNG 147 KB)

Supplementary file3 (PNG 743 KB)

## Data Availability

The authors confirm that the data supporting the findings of this study are available within the article and its supplementary materials.
