# Supplementary material for: Computational fluid dynamics for vascular assessment in hepatobiliopancreatic surgery: a pilot study and future perspectives
Source: Surg Endosc. 2025 Apr 1;39(5):3127–36. doi: 10.1007/s00464-025-11536-4 (PMC12041174; doi:10.1007/s00464-025-11536-4)
Supplement: Supplementary file 1 — Supplementary file1 (DOCX 20 KB) [file 464_2025_11536_MOESM1_ESM.docx]

| **ID** | **CHA flow (ml/min)** | **GDA flow (ml/min)** | **CHA1 (clamped GDA) (ml/min)** | **GDA1 (clamped CHA) (ml/min)** |
| --- | --- | --- | --- | --- |
| **ID1** | 374 | -155 | 344 | 104 |
| **ID2** | 112 | -49 | 98 | 6 |
| **ID3** | 244 | -90 | 209 | 92 |
| **ID4** | 257 | -94 | 458 | 100 |
| **ID5** | 246 | -82 | 234 | 104 |
| **ID6** | 374 | -155 | 344 | 104 |
| **ID7** | 465 | -93 | 390 | 63 |
| **ID8** | 226 | -78 | 156 | 78 |
| **ID9** | 125 | -110 | 85 | 105 |
| **ID10** | 179 | -52 | 262 | 203 |
| **ID11** | 133 | -15 | 112 | 22 |
| **ID12** | 302 | -95 | 219 | 29 |
| **ID13** | 209 | -54 | 163 | 121 |
| **ID14** | 214 | -69 | 209 | 92 |
| **ID15** | 385 | -70 | 347 | 99 |

**Table S1.** Intraoperative blood flow rate measurement of patient data used for computational model development. Negative GDA flow represents GDA flow from CHA to the pancreas. CHA1 shows common hepatic artery flow after GDA clamping. GDA1 shows gastrodudodenal artery flow after CHA clamping. Abbreviations: ID; identification number, GDA; gastroduodenal artery, CHA; common hepatic artery.
