# Supplementary figures and images for: Computational fluid dynamics for vascular assessment in hepatobiliopancreatic surgery: a pilot study and future perspectives
Source: Surg Endosc. 2025 Apr 1;39(5):3127–36. doi: 10.1007/s00464-025-11536-4 (PMC12041174; doi:10.1007/s00464-025-11536-4)

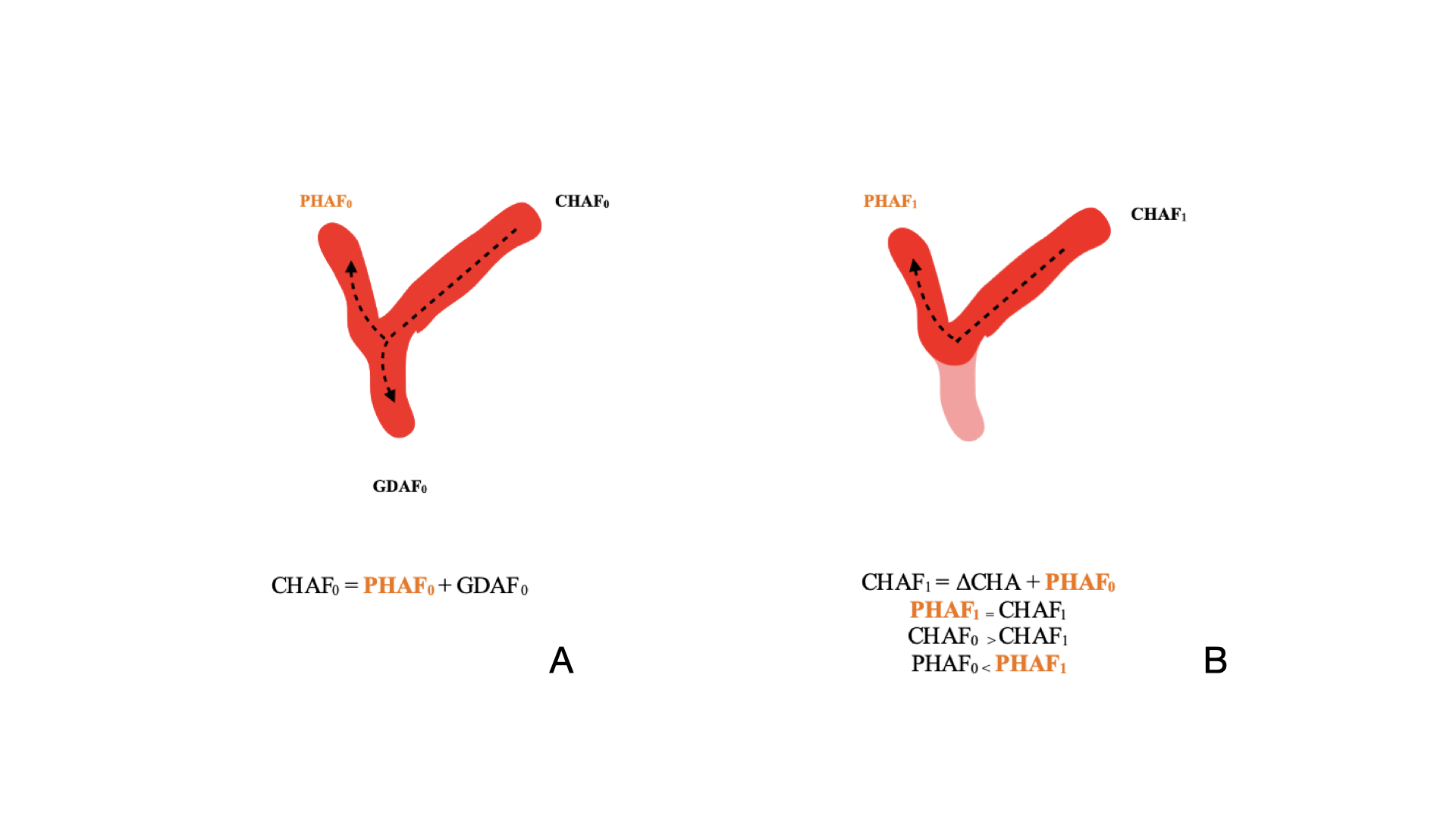

Supplement: Supplementary file 2 — Supplementary file2 (PNG 147 KB) [file 464_2025_11536_MOESM2_ESM.png]

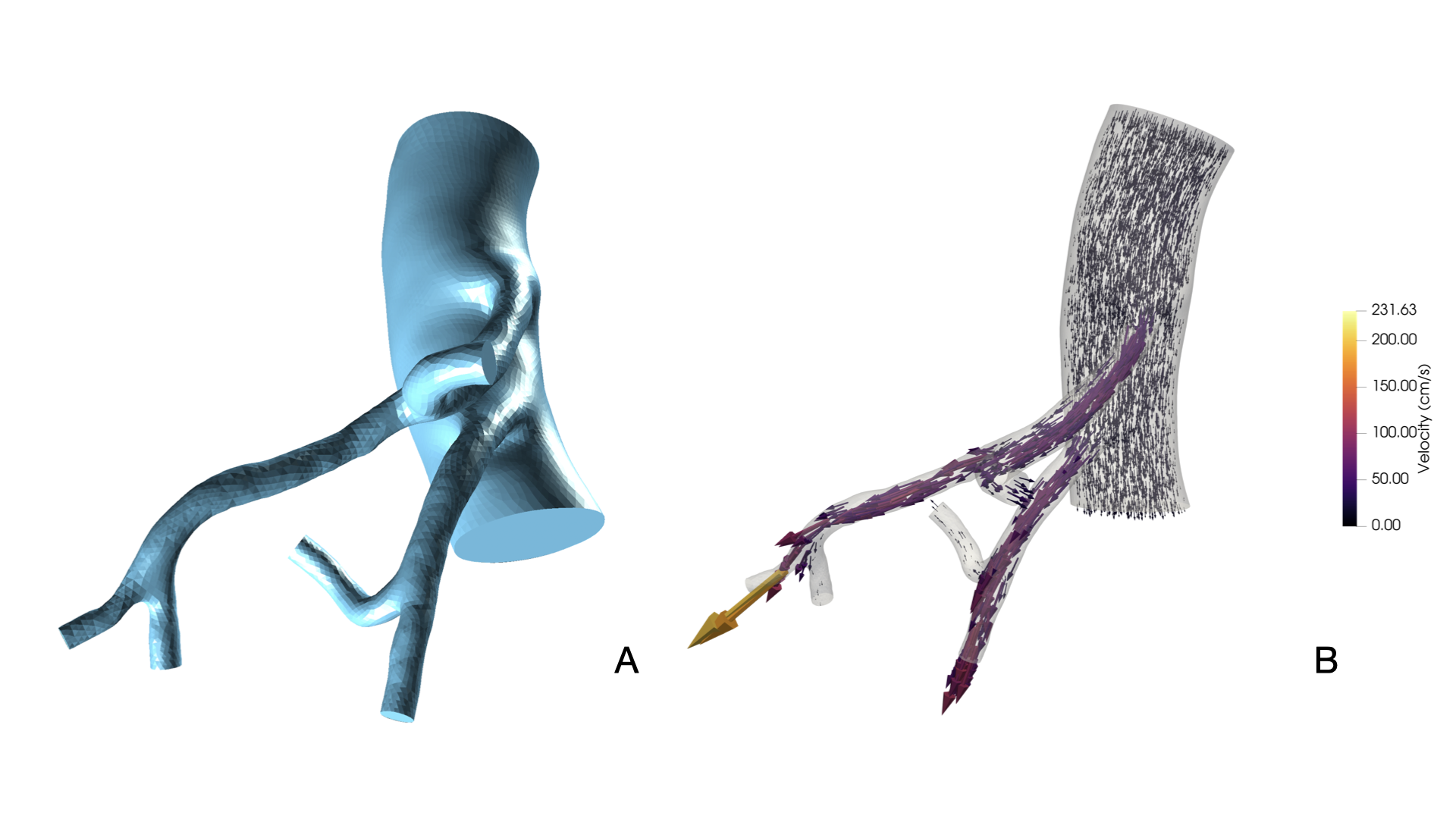

Supplement: Supplementary file 3 — Supplementary file3 (PNG 743 KB) [file 464_2025_11536_MOESM3_ESM.png]
